# Face-Computer Interface (FCI): Intent Recognition Based on Facial Electromyography (fEMG) and Online Human-Computer Interface With Audiovisual Feedback

**DOI:** 10.3389/fnbot.2021.692562

**Published:** 2021-07-16

**Authors:** Bo Zhu, Daohui Zhang, Yaqi Chu, Xingang Zhao, Lixin Zhang, Lina Zhao

**Affiliations:** ^1^State Key Laboratory of Robotics, Shenyang Institute of Automation, Chinese Academy of Sciences, Shenyang, China; ^2^Institutes for Robotics and Intelligent Manufacturing, Chinese Academy of Sciences, Shenyang, China; ^3^University of Chinese Academy of Sciences, Beijing, China; ^4^Rehabilitation Center, Shengjing Hospital of China Medical University, Shenyang, China

**Keywords:** face-computer interface, facial electromyography, facial movements, robotic arm control online, rehabilitation assistance robot

## Abstract

Patients who have lost limb control ability, such as upper limb amputation and high paraplegia, are usually unable to take care of themselves. Establishing a natural, stable, and comfortable human-computer interface (HCI) for controlling rehabilitation assistance robots and other controllable equipments will solve a lot of their troubles. In this study, a complete limbs-free face-computer interface (FCI) framework based on facial electromyography (fEMG) including offline analysis and online control of mechanical equipments was proposed. Six facial movements related to eyebrows, eyes, and mouth were used in this FCI. In the offline stage, 12 models, eight types of features, and three different feature combination methods for model inputing were studied and compared in detail. In the online stage, four well-designed sessions were introduced to control a robotic arm to complete drinking water task in three ways (by touch screen, by fEMG with and without audio feedback) for verification and performance comparison of proposed FCI framework. Three features and one model with an average offline recognition accuracy of 95.3%, a maximum of 98.8%, and a minimum of 91.4% were selected for use in online scenarios. In contrast, the way with audio feedback performed better than that without audio feedback. All subjects completed the drinking task in a few minutes with FCI. The average and smallest time difference between touch screen and fEMG under audio feedback were only 1.24 and 0.37 min, respectively.

## 1. Introduction

Patients with paralysis and amputation are usually accompanied by loss of limb motor function. Particularly, for the patients with upper limb amputation, high paraplegia, or muscle weakness, it is hard to take care of themselves due to the loss of partial or total motor functions of hands or feet. In order to restore the patient's lost limb function or assist them for daily activities such as eating and drinking, artificial hands, exoskeletons, robotic arms, smart wheelchairs and other assistive robots have emerged (Wu et al., 2018; Kaur, 2021). How to establish a natural, efficient and stable human-computer interface (HCI) has become a difficult and hot point in the research of interactive control of rehabilitation aids (Mussa-Ivaldi et al., 2013; Venkatakrishnan et al., 2014; Gordleeva et al., 2020; Xiong et al., 2021).

Traditional HCI methods such as those based on buttons, joysticks, or touch screen are usually no longer applicable due to lack of limb function in the above-mentioned situations. In order to solve these problems and optimize the HCI of rehabilitation and assistive machines, many researchers have begun to study HCI based on human physiological signals such as electroencephalogram (EEG), surface electromyography (sEMG), electrooculography (EOG), and so on (Shin et al., 2017; Ding et al., 2019; Zhang et al., 2019; Gordleeva et al., 2020; Li et al., 2020). Compared with lower recognition accuracy or need additional stimulation for EEG-based HCI (such as motor imagery and steady state visual evoked potential) (Lin et al., 2016; Chu et al., 2018) and relative fewer recognizable intentions for EOG-based HCI (Bastos-Filho et al., 2014; He and Li, 2017) or hybrid gaze-brain machine interface (Li et al., 2017; Krausz et al., 2020; Zeng et al., 2020), EMG-based HCI has been widely used in the field of neurorehabilitation with the advantage of higher accuracy and stability, especially for decoding motor intentions of the limb with EMG (Ding et al., 2015; Hussain et al., 2016; Zhang et al., 2019). However, intent recognition based on limb EMG is still facing a huge challenge due to the abnormal signal in the absence of limb function (Jaramillo-Yánez et al., 2020; Xiong et al., 2021). Hence, instead of limb EMG, a novel intention recognition method based on facial electromyography (fEMG) and the HCI based on fEMG have been paid attention and partly researched (Hamedi et al., 2011; Tamura et al., 2012; Bastos-Filho et al., 2014; Nam et al., 2014; Inzelberg et al., 2018; Kapur et al., 2018, 2020).

There are many muscles on the human face, which can control different parts of the face to produce many different movements or expressions, such as eyebrows, eyes, lips, teeth, and so on. Thus, rich information can be decoded from fEMG signals (Hamedi et al., 2013; Inzelberg et al., 2018). Hamedi et al. (2011) recognize movement intentions from fEMG, and a total of 11 facial movements were recognized through electrodes attached to the forehead, with an accuracy rate of over 90%. In their work, a multipurpose interface was suggested that can support 2–11 control commands that could be applied to various HMI systems. Kapur et al. (2020, 2018) developed a portable and wearable device to collect EMG signals around the mouth and neck. More than 10 speech or silent voice commands were recognized from the collected EMG signal. Lu and Zhou (2019) used three electrodes to collect fEMG around the mouth to recognize five movements and used them to control the cursor on the computer to complete functions such as drawing and typing. Cler and Stepp (2015) developed a system using fEMG typing, and the system's typing ITR reached 105.1 bits/min. Nam et al. (2014) integrated multiple signals such as EOG and fEMG to control a humanoid robot. Zhang et al. (2020) controlled a two-degree-of-freedom (2-DOF) prosthesis based on fEMG. Bastos-Filho et al. (2014) and Tamura et al. (2012) used fEMG to control the movements of a wheelchair.

Although there have been some studies on the use of fEMG to recognize intentions to realize HCI, there are still many unsolved problems in this field. First of all, most of the current researches are only based on an organ of the human face, such as the actions of the mouth only or the movements of the eyes only (Hamedi et al., 2011; Lu and Zhou, 2019). Therefore, the performance of fusion of forehead, eyes, mouth, and other parts needs further research. What's more, the facial muscles are neither intertwined like the muscles that control the fingers of the forearm, nor are they independent of each other like the muscles of the upper arm and thigh. There are few research on which features and models are suitable for fEMG classification and recognition. In addition, most of the existing researches only control cursors or mobile robots (Tamura et al., 2012; Bastos-Filho et al., 2014; Nam et al., 2014; Cler and Stepp, 2015), and there are few researches using fEMG to control interactive device with human such as robotic arms. In particular, there is a lack of experiments to control the robotic arm to assist users in completing daily tasks such as eating or drinking based on fEMG. What's more, most of the existing studies only have visual feedback, and lack other feedback methods such as auditory feedback.

In order to solve these problems, we conducted detailed research and experiments. In this study, a complete face-computer interface (FCI) framework based on fEMG including offline analysis and online control of mechanical equipments was proposed. This is a limbs-free method, thus patients can use it to control prostheses, exoskeletons, robotic arms, and computers to take care of themselves and communicate with the world. Healthy people can also use it as a third way of interaction, for example, when controlling an intelligent robotic arm, they can use it as a third hand. In our research, six facial movements related to eyebrows, eyes, and mouth were used in FCI. In order to select better models and features for online FCI, 12 models, eight ways of calculating features, and three different feature combination input methods for the model were compared in detail in the offline stage. In the online stage, four well-designed sessions were designed to control a robotic arm to complete drinking water task in three ways (by touch screen, by fEMG with, and without audio feedback) for verification and performance comparison of proposed FCI framework. To our best of knowledge, this is the first study of using fEMG to control a robotic arm to complete a drinking experiment with audiovisual feedback.

In summary, the main contributions and highlights of this research are as follows: (1) A complete Face-Computer Interface (FCI) framework based on fEMG has been proposed. At the same time, the effectiveness of FCI has been proven through well-designed experiments. (2) The performance of frames with and without audio feedback was compared. (3) Multiple models and features were carefully compared and analyzed to select the best model and features suitable for FCI.

The rest of this article would introduces the concept of the FCI framework first, and then six facial movements, eight features, 12 models and three different feature combination methods for model inputing were explained immediately after. The offline data acquisition with its analysis and four online experiment sessions to complete drinking water task based on different ways were written in detail after that. Detailed experimental results and discussion were introduced at the end.

## 2. Methods

### 2.1. Frame of FCI

**Figure 1:**
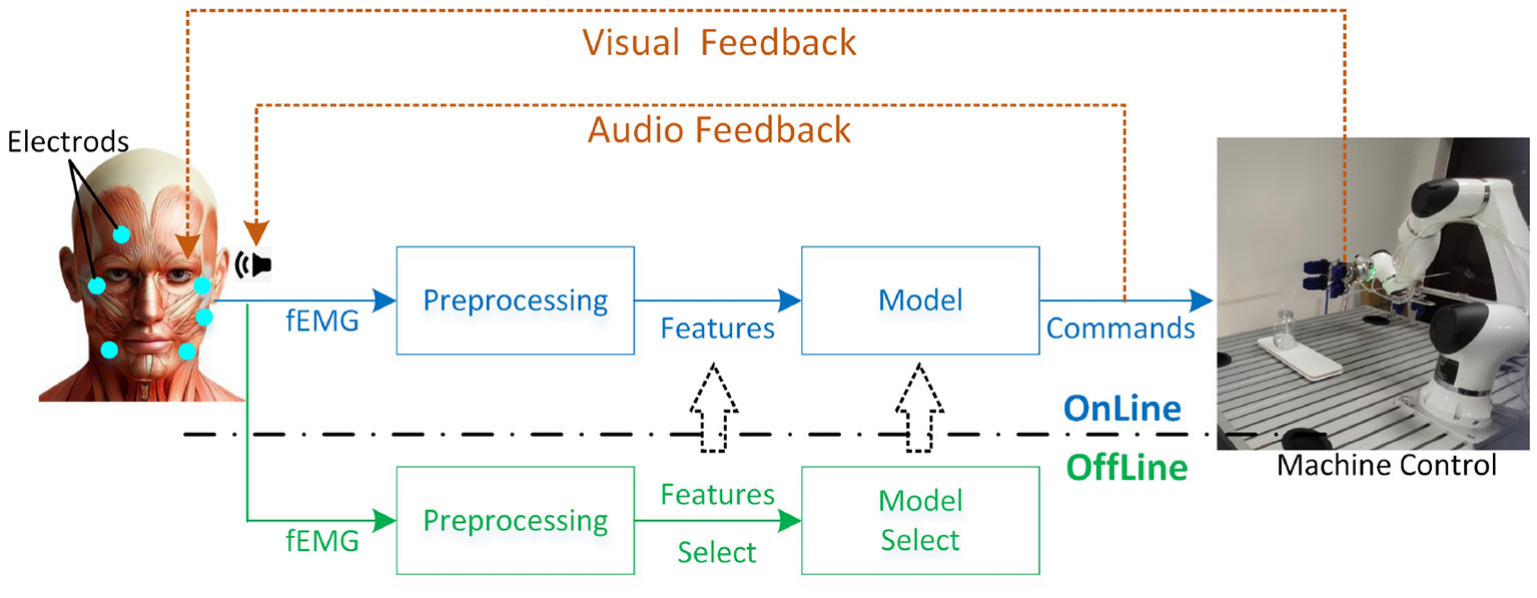
The frame of the face-computer interface (FCI).

As shown in Figure 1, the purpose of the FCI framework is to use fEMG for online control of specific machines such as exoskeleton, prosthetic hand, robotic arm, computer application, and so on. The fEMG is acquired by some electrodes attached on human's face. Then fEMG signals will go through offline preprocessing, feature extraction, and model selection to determine the appropriate features and model for online control stage. After a suitable online model is selected, fEMG will undergo the same filtering and other preprocessing as when offline. The commands or signals output by the model are used to control the machine which is a robotic arm with a soft grip in our research. As usual, subjects can watch the movement of the controlled device in real time to provide visual feedback. In addition, we have also added a voice broadcast of the output commands of the model to provide more feedback information to the users.

### 2.2. Facial Movements

**Figure 2:**
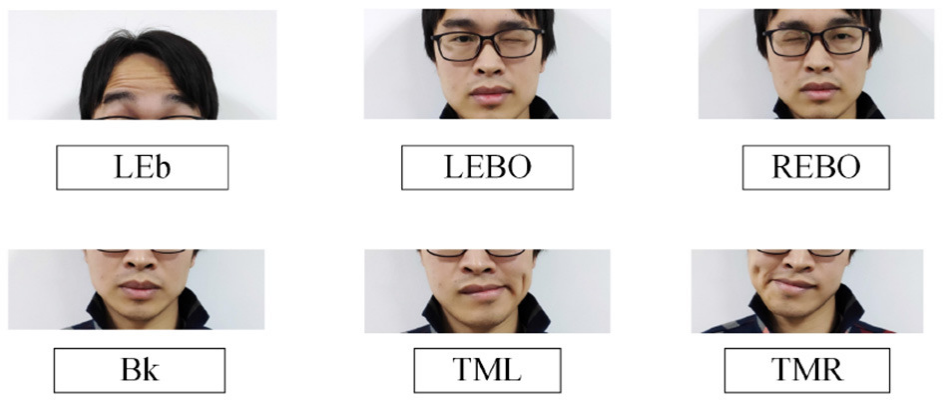
Illustration of facial movements. From left to right and from top to bottom are Lift Eyebrows (LEb), Left Eye Blink Once (LEBO) slowly, Right Eye Blink Once (REBO) slowly, Bick (Bk), Tilt Mouth to Left (TML), and Tilt Mouth to Right (TMR).

For the purpose to study FCI based on fEMG in this paper, almost the entire facial areas are involved as the research object, including the forehead area related to eyebrows movements, the area around the eyes related to eyelid actions, and the area related to mouth movements. In order to make the selected actions can be performed by most participants, six movements selected elaborately are used for research. As shown in Figure 2, those movements include Lift Eyebrows (LEb), Left Eye Blink Once (LEBO) slowly, Right Eye Blink Once (REBO) slowly, Bick (Bk), Tilt Mouth to Left (TML), Tilt Mouth to Right (TMR), and REST of course. The LEb requires participants to raise the left and right eyebrows at the same time. The Bk is like simulating stationary chewing a hard food. Subjects need to consciously blink the corresponding eye slowly (close corresponding eye for more than hundreds of milliseconds) when doing the LEBO or the REBO movements. Participants can choose to shift only the corner of the mouth to the left or both the corner of the mouth and the mandible to the left according to their personal habits when performing the TML. Actually, the requirement for performing the TMR is same as the TML. And the REST requires participants relaxation and doing nothing.

### 2.3. Features and Models

**Figure 3:**
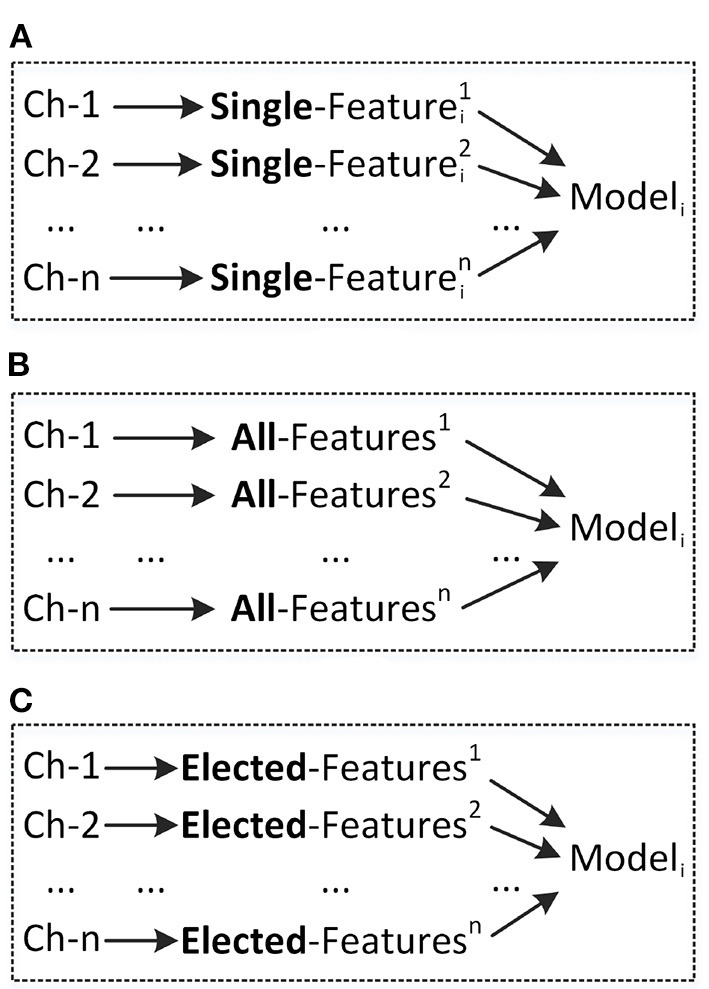
Different combinations of channels, features, and models. **(A)** Using single feature (SF) as model input. **(B)** Using all features (AF) as model input. **(C)** Using elected features (EF) as model input.

In order to determine a more suitable model and features for on-line control machines in FCI frame, 12 models and eight types of feature were compared in detail, respectively. The 12 models are {Logistic Regression (LR), Naive Bayes (NB), Decision Tree (DT), Support Vector Machines (SVM) with Linear Kernel, Multilayer Perceptron (MLP), Ridge Classifier (Ridge), Random Forest (RF), Quadratic Discriminant Analysis (QDA), Ada Boost (Ada), Gradient Boosting Classifier (GBC), Linear Discriminant Analysis (LDA), Light Gradient Boosting Machine (LGBM)} (VanderPlas, 2016; Jaramillo-Yánez et al., 2020). Furthermore, as shown in Figure 3, three different feature combination methods for model inputing were analyzed in detail, which are Single-Feature (SF) per channel, All-Features (AF), Elected-Features (EF). Notably, the features in EF were selected according to the order of performance of SF.

Assume that *x*_*i*_ is the *i*-th point in an EMG signal with *N* points. The calculation method of each feature is as follows (Roberto and Dario, 2016; Jaramillo-Yánez et al., 2020).

(1) Mean absolute value, MAV

$$MAV=\frac{1}{N}\sum_{i=1}^{N}\left|x_{i}\right|$$

(2) Root mean square, RMS

$$RMS=\sqrt{\frac{1}{N}\overset{N}{\underset{i=1}{\sum }}x_{i}^{2}}$$

(3) Mean changes, MC

$$MC=\frac{1}{N}\overset{N-1}{\underset{i=1}{\sum }}\left(x_{i+1}-x_{i}\right)$$

(4) Mean absolute changes, MAC

$$MAC=\frac{1}{N}\sum_{i=1}^{N-1}\left|x_{i+1}-x_{i}\right|$$

(5) Maximum value, MAX

$$MAX=max\left(x_{i}\right),i=1,2,...,N$$

(6) Zero crossings, ZC

$$ZC=\sum_{i=1}^{N-1}sgn(-x_{i}x_{i+1})$$

Where the *sgn*(ζ) represents the sign of ζ

$$sgn(\zeta )=\{\begin{array}{ll} 1 & \zeta >0\\ 0 & other \end{array}$$

(7) Variance, VAR

$$VAR=\frac{1}{N-1}\overset{N-1}{\underset{i=1}{\sum }}{\left(x_{i}-\bar{x}\right)}^{2}$$

(8) Auto regression coefficient, ARC

$$x_{i}=\overset{p}{\underset{k=1}{\sum }}a_{k}x_{i-k}+e_{i}$$

Where the coefficients *a*_*k*_ are the features. The *p* is the order of ARC which is three in this study.

## 3. Experiment

### 3.1. Subjects and Devices

A total of seven healthy subjects (2 females, 26.2 ± 5.2 years old) participated in all experiments in this study. And it was reviewed and approved by the Ethical Committee of the Shenyang Institute of Automation. Before starting all processes such as data collection and robotic arm control, all subjects were informed in detail of all experimental procedures and possible dangers, and signed an informed consent form.

Four main types of hardware devices [a fEMG collector, a robotic arm, a personal computer (PC), and a high-speed router] were used in this study. The NeusenW64 (Neuracle Co., Ltd, China) with a maximum of 64 unipolar channels (or 32 channels of bipolar electrodes) and maximum of 2,000 Hz sampling rate was used for the acquisition of fEMG. The fEMG collector used wifi to transmit data to the PC in real time through a high-speed wireless router. The PC containing with 64-bit Windows-10 system and Python3.7 programming environment was used for all data acquisition, data analysis, intent recognition model training, and control of the robotic arm (Elfin, Han's Robot Co., Ltd, China) with a soft gripper.

All fEMG acquisition processes used 1,000 Hz sampling rate to acquire signals. After the original signal was acquired, the signals used for further analysis were preprocessed by IIR notch filtering to remove power frequency interference and 10–450 Hz second-order bandpass Butterworth filtering.

### 3.2. fEMG Acquisition for Offline Analysis

#### 3.2.1. Electrodes Configuration

In order to collect the fEMG signals corresponding to different actions, six monopolar electrodes were attached to different parts of the human face. The labels, positions, and corresponding muscles of all electrodes are shown in the Table 1. The No. 1 electrode was placed on forehead for acquainting fEMG signals when LEb being performed. Electrodes No. 2 and No. 3 were placed on the extended corners of the left and right eyes, respectively to collect the fEMG signals generated by the actions of LEBO and REBO. When the subjects clenched their teeth, the position of the masseter muscle would be obviously raised, and the No. 4 electrode was placed on the corresponding raised part of the left face. Electrodes No. 5 and No. 6 were placed on the left and right corners of the mouth to collect the fEMG signals generated by the actions of TML and TMR, respectively. In order to balance the electrical field of the left face and the right face, two short-circuited reference electrodes were placed on the mastoid behind the left and right ears. The ground electrode was placed behind the right ear and next to the reference electrode. All electrodes are ordinary disposable electrodes with conductive paste. Before applying the electrodes, the subject's face was wiped with alcohol and waited for it to dry. Each numbered electrode was placed according to the structural features of the human face, such as the corners of the eyes, the edge of the mandible, the corners of the mouth, etc., to prevent large positional deviations from the collection of different sessions.

**Table 1:** Correspondence of electrode numbers, positions, muscles, movements, and commands.

**Numbers**	**Positions**	**Muscles**	**Movements**	**Commands**
1	Forehead	Frontal muscle	LEb	Positive direction
2	Left eye corner	Left orbicularis oculi	LEBO	Y axis (forward or back)
3	Right eye corner	Right orbicularis oculi	REBO	X axis (left or right)
4	Masseter skin	Masseter	Bk	Negative direction
5	Left corner of mouth	Left risorius	TML	Gripper (open or close)
6	Right corner of mouth	Right risorius	TMR	Z axis (up or down)

#### 3.2.2. Acquisition Paradigm

For the six actions {LEb, LEBO, REBO, Bk, TML, TMR} defined in Figure 2, each subject participated in 25 rounds of data collection, and each action was collected once in a round. In order to familiarize the subjects with the collection process and maintain the consistency of their actions, the first 5 rounds were used as adaptive training. The next 20 rounds were for the formal collection of fEMG data. In the entire collection process, each action was executed 25 times in total, of which the fEMG signals generated by the last 20 actions were regarded as valid signals. After the first five rounds of adaptive training were completed, the subjects had 3 min of rest and then started the formal collection. During each round of collection, the order of appearance of the six actions was random. There was a 5 s rest between the two actions, and each action lasted about 3 s. Before a action was executed, the name prompt and voice prompt of the action were given by screen and a speaker at the same time. Participants rested for 1 min after each round. After the formal collection of 10 rounds, the subjects rested for 5 min and then performed the next 10 rounds of collection. The entire collection time lasted ~1 h.

#### 3.2.3. Offline Data Analysis and Processing

**Figure 4:**
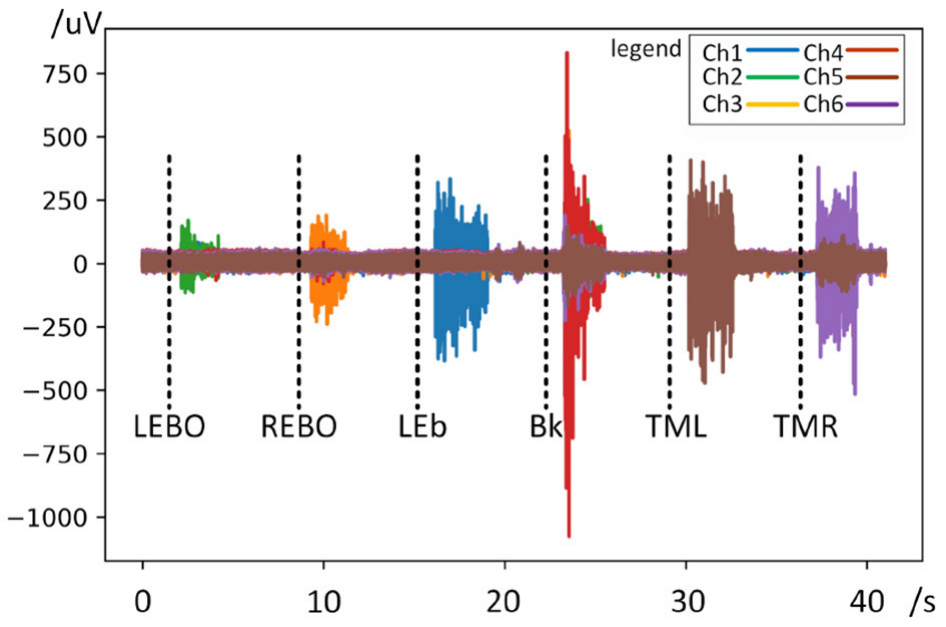
Waveform diagram of a round in data acquisition. The black dotted lines indicate the action prompt.

**Figure 5:**
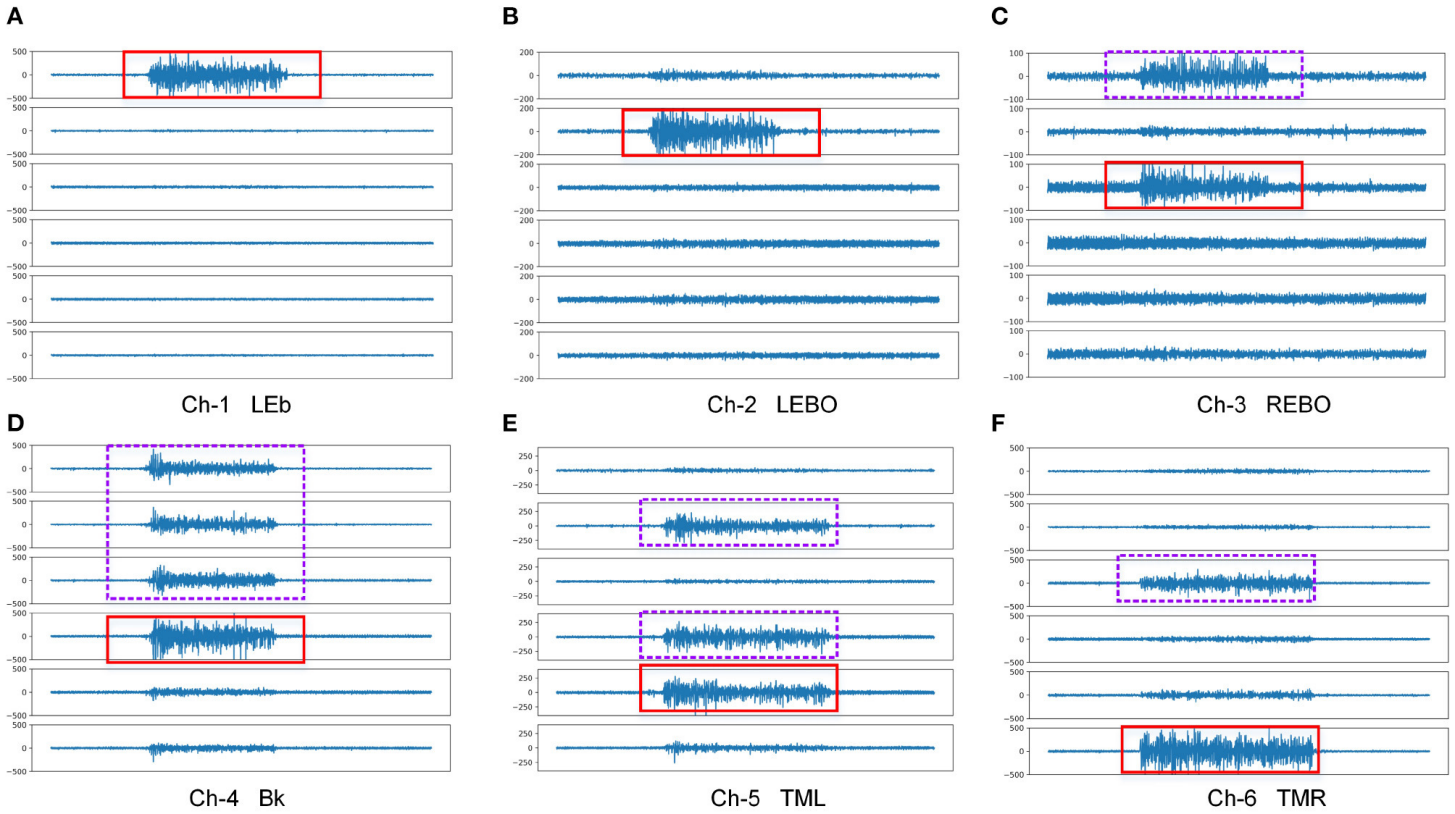
Waveform comparison between different actions and channels. In the figure, the waveform in the solid red frame is the main channel signal corresponding to the action, and the waveform in the purple dashed frame is redundant signal collected by other channels. **(A)** Ch-1 LEb, **(B)** Ch-2 LEBO, **(C)** Ch-3 REBO, **(D)** Ch-4 Bk, **(E)** Ch-5 TML, **(F)** Ch-6 TMR.

Figure 4 shows a demo of a round of waveform in data acquisition and Figure 5 shows a demo of waveform comparison between different actions and channels. It can be seen from the waveform that the fEMG signal corresponding to each action has an obvious difference. The signal amplitudes from LEBO and REBO are obviously smaller than those of the other actions, while the amplitude from Bk is the largest, followed by LEb. Thus, we cannot simply identify the action category from the amplitude of each signal because of the mutual influence between the channels as shown in Figure 5. In the Figure 5, the waveform in the solid red frame is the main channel signal corresponding to each action, and the waveform in the purple dashed frame is redundant signal collected by other channels. The action Bk has an effect on almost all channels, while the LEb has almost no effect on other channels. Almost all channels have an influence from actions that are not their counterparts. Ch-1 is affected by REBO because it is attached to the above of the right eye, and of course it is also affected by Bk. Ch-2 is affected by Bk and TML and similarly Ch-3 is affected by Bk and TMR. Ch-4 is affected by TML for Ch-4 is attached at left face. Ch-5 and Ch-6 are the least affected, but are also partially affected by Bk.

Since the intent could not be easily identified from fEMG signal, the features and models mentioned in the third subsection of the second section were used to identify the action category and were analyzed in detail at offline. A 200 ms sliding window with a 50 ms sliding interval containing fEMG signals was used to calculate each feature. For each channel of each action, the signal between 1,500 and 2,650 ms after the prompt was divided into 20 samples. Similarly, the 350 ms signal from 350 ms before each prompt to the prompt moment was divided into four samples. In this way, for a round of collecting 6 actions, there were 20 samples for each movement and 24 samples for REST. To sum up, there were actually 2,880 samples for each participant (2,880 = 20 rounds * 20 samples of each round * 6 actions + 20 rounds * 24 REST samples of each round). Five-fold cross-validation was used to analyze the performance of each model for each subject. For each cross-validation, 80% of the data (2,304 samples = 6 actions * 320 samples for each action + 384 samples for REST) was used for training, and the remaining 20%(576 samples) was used for testing. In other words, 320 samples of each action were used for training in each cross-validation, and the remaining 80 samples were used to test the performance of trained model.

### 3.3. Control Robotic Arm Online

**Figure 6:**
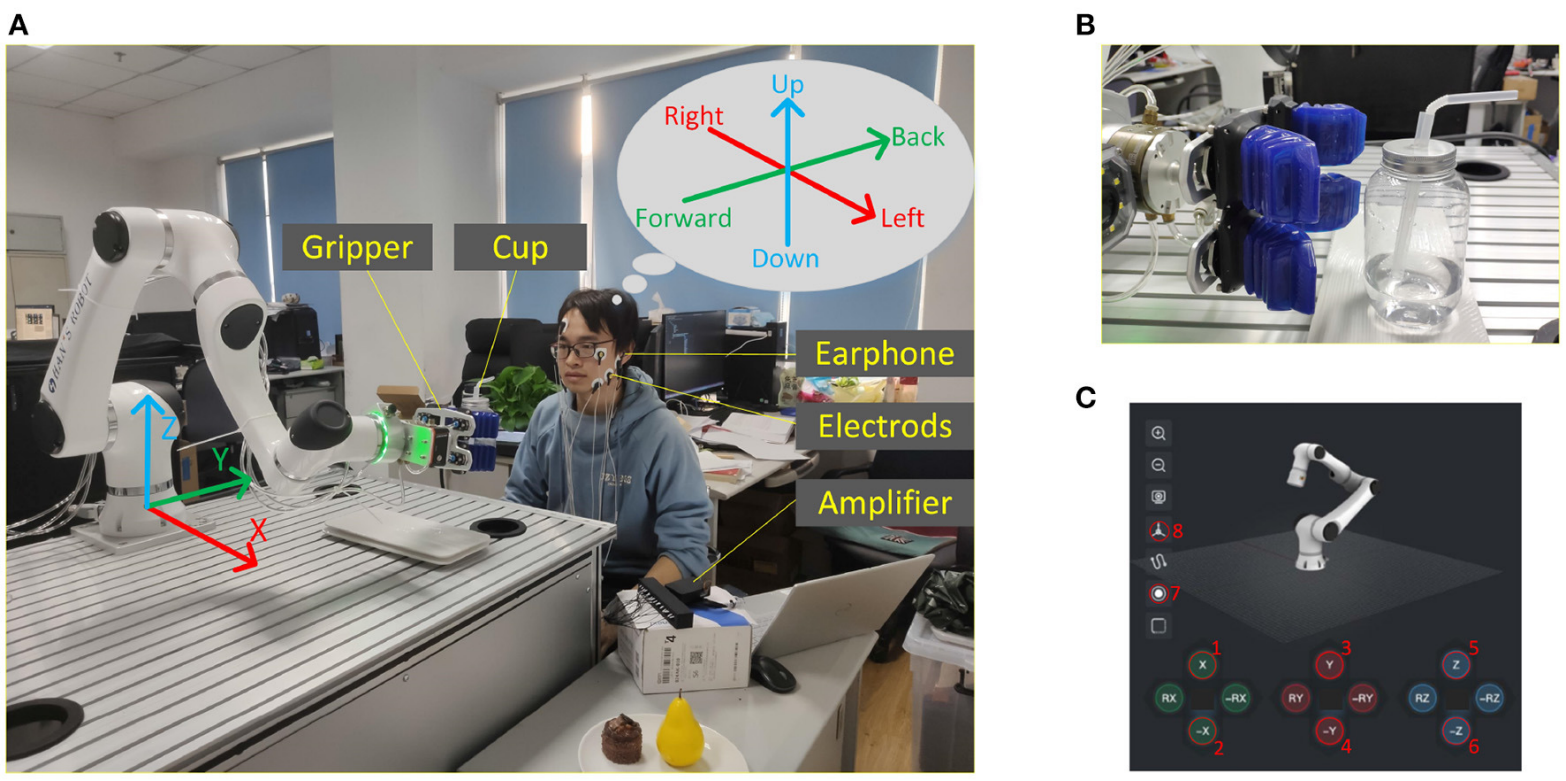
Online control of robotic arm through fEMG. **(A)** A snapshot of the online control of the robotic arm to drink water. The robotic arm body and its coordinate system, the soft gripper, fEMG electrodes, earphone for audio feedback, amplifier, PC, etc. are marked. **(B)** A photo of the soft gripper before grabbing the cup. **(C)** Virtual button interface for manipulating the robotic arm.

In order to simulate the scenario where FCI is used in the environment of lack of limb function, a water drinking task was carefully designed. Participants were required to complete the task of using fEMG to control the robotic arm to drink water for themselves during the online control period. A six-degree-of-freedom robotic arm was controlled in this phase and Figure 6 shows the details of this scene. A soft gripper mounted on the end of the robotic arm was used to grab the object as shown in Figures 6A,B. The soft gripper was driven by a dynamic driving manner so as not to damage the object while grasping the object. A drinking glass with a diameter of 7.5 cm and a height of 13 cm with a straw was used for the drinking experiment.

In the experiment, the robotic arm can be controlled in two ways. One of them is to control through the teach pendant which has a touch screen. On the touch screen of the teach pendant, when the robot arm is controlled in manual control mode, a virtual button interface that can control the motion of the robot arm is presented as shown in Figure 6C. One can control the movements of the robotic arm corresponding to the function of virtual button by pressing and holding the virtual button on the touch screen. Another way to control the robotic arm is through the FCI method proposed in this paper.

During the whole experiment, the robotic arm works under the position control mode of Cartesian space. The three-dimensional orthogonal Cartesian coordinate system is shown in Figure 6. In this research, the external application sends eight commands to the robotic arm through the API of the robotic arm system. These eight commands are {moving to the positive of X-axis, moving to the negative of X-axis, moving to the positive of Y-axis, moving to the negative of Y-axis, moving to the positive of Z-axis, moving to the negative of Z-axis, closing gripper, and opening gripper}. These eight commands correspond one-to-one with the virtual buttons labeled 1–8 in Figure 6C. When the robotic arm is working, the operator only needs to pay attention to the direction of movement of the claw at the end of the manipulator, and does not need to pay attention to the joint space of the manipulator. The mapping from Cartesian space to joint space is done by the manipulator API. For the safety of operation, the end of the robot arm runs at a lower speed of 3 cm/s during the movement. In this experiment, the working space of the gripper is limited to a cuboid space with a range of [20, 120] cm of X-axis, [−70, 100] cm of Y-axis, and [5, 120] cm of Z-axis. The projection of the working space at the end of the robotic arm on the X-Y plane is restricted to not exceed the desktop as shown in Figure 6A, except for the side parallel to X-axis at positive direction of Y-axis, which can exceed 30 cm so that the water cup can be sent to the subject's mouth.

As the subjects were naive for controlling robotic arm, the experiment was divided into four sessions. Subjects participated in different sessions for 4 consecutive days, and each session took from 1 to 2 h. The first session was for participants to familiarize themselves with the robotic arm and its control process. In the second session, the participants used virtual buttons to operate the robotic arm to complete the task of drinking water. In the third and fourth sessions, subjects used FCI to complete the task of drinking water with and without audio feedback, respectively.

#### 3.3.1. Session #1-Familiar With the Robotic Arm

In this session, participants' goal was to understand the movement of the end of the manipulator in the robotic arm workspace and then use virtual buttons to control the gripper of the robotic arm for single-axis movement. A professional robotic arm engineer explained to each participant the working space of the robotic arm and the uniaxial movement of the end of the robotic arm in the Cartesian coordinate system. In this process, participants did not need to understand the working principle of the robotic arm such as joint angle, joint space, kinematics, etc., but only need to know that the gripper can move along the three axes in Cartesian space. They even did not need to know the concept of Cartesian space. The instructor explained to them as follows: “The gripper will move forward when this button (−Y) is pressed. And the gripper will move to the left when this button (+X) is pressed. After that button (Close) is pressed, the gripper will close.” After understanding the operating mode of the end of the robotic arm, the subjects used eight virtual buttons on the touch screen to control the direction, opening or closing of the gripper. Each participant was asked to run every control command 10–15 times in order to become familiar with the movement of the gripper. For each subject, this session lasts about 1 h.

#### 3.3.2. Session #2-Complete Drinking Task Based on Virtual Buttons

In this session, subjects were asked to use virtual buttons to control the movement of gripper to complete drinking task 10 times. In each drinking task, as shown in Figure 6, the cup was placed on a 30*15 cm rectangular saucer. The center of the saucer was fixed at the position (x: 90, y: 45, z: 0) cm of the robotic arm coordinate system. In order to ensure that the subjects were controlled based on their actual location rather than remembering repeated paths, in each task, the position of the cup on the saucer and the initial position of the gripper were random. At the same time, in order to eliminate the difference in experimental performance caused by randomness, the random range of the position of the cup and the gripper was restricted rather than completely random, and each participant conducts at least 10 experiments in each session. The initial position of the gripper was (x: *x*_*i*_, y: -45, z: *z*_*i*_)cm, *i* = 1, 2, 3, ..., 10, in each drinking task. The *x*_*i*_ was randomly generated from [20, 120] cm and *z*_*i*_ was randomly generated from [5, 120] cm. The subject sat in the direction of the positive Y-axis and was asked to use virtual buttons to control the gripper to complete the task of drinking water. A water drinking task can be divided into three stages: (1) the gripper was moved from a random initial position to the place where the water cup was placed; (2) the water cup was picked up and moved to the subject's mouth; (3) the subject was asked to drink a small amount of water and then controlled the robotic arm to place the water cup back to the saucer. The time spent on each task was recorded when the gripper started to move, and stopped when the water cup was put back on the saucer. The subjects needed to complete 10 repeated drinking tasks during this session and there was a 2 min rest period between each task, during which the experiment assistant would reset the water cup and the gripper to the initial position.

#### 3.3.3. Session #3 and #4-Complete Drinking Task Based on FCI

**Figure 7:**
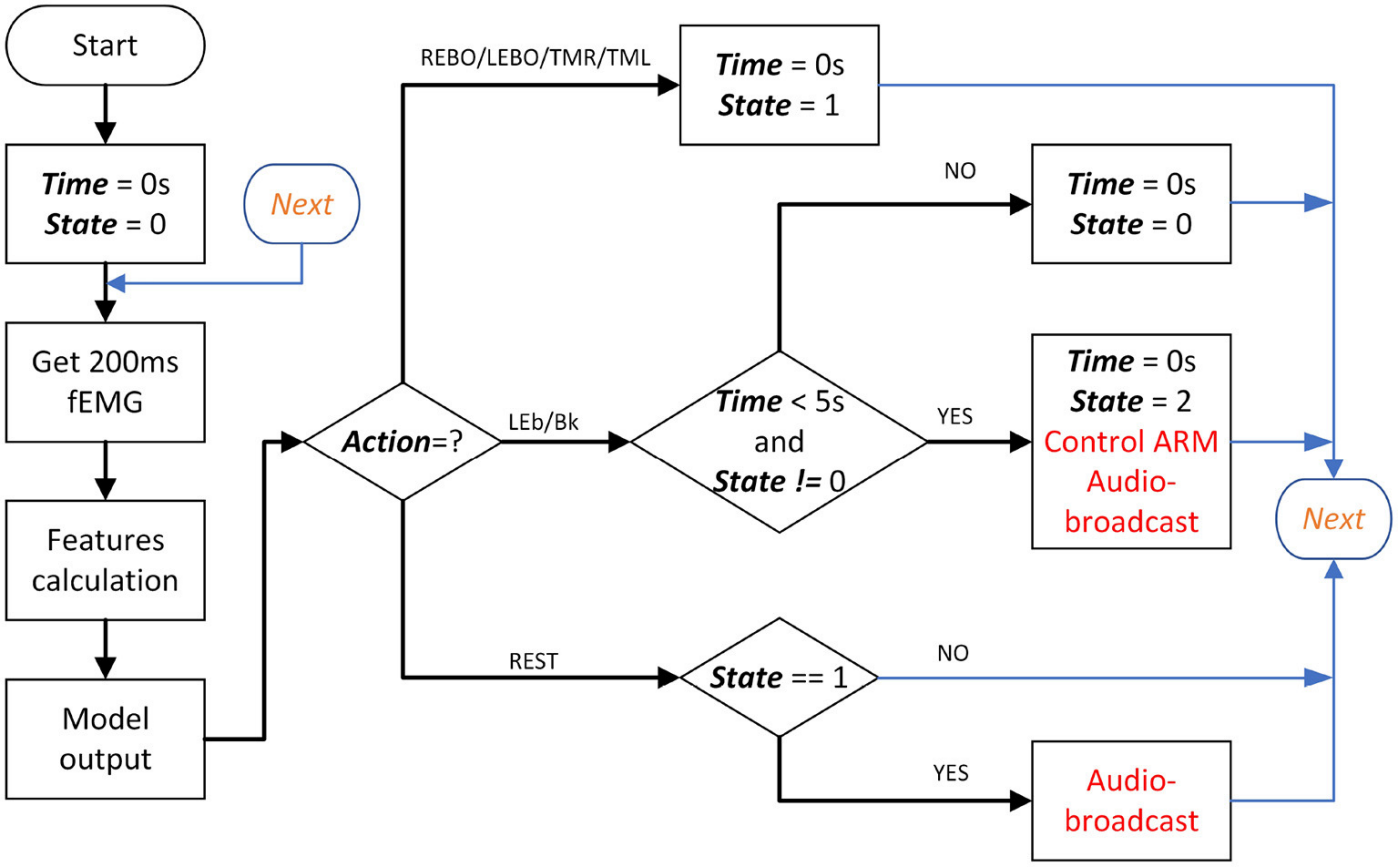
Online control flow chart base on FCI. In the figure, the black, bold, and italicized words are the main variables, and the red part is the robotic arm control and voice broadcast.

In this two sessions, participants used fEMG instead of virtual buttons to control gripper to complete the task. Except for the change of the command input interface, the other settings remained the same as in the second session. The models and features selected during the offline phase were used here. Before this two sessions started, in order to eliminate the difference between the different sessions, the subjects were asked to collect five additional rounds of fEMG data to update the model with the initial parameters trained in the offline phase. When fEMG was used as a control method, six facial movements were mapped into eight commands corresponding to eight gripper movements. As shown in Table 1 and Figure 7, each manipulator motion control goes through two stages. The first stage is to use four actions to select one of the four axes (3 coordinate axes and hand grip to open or close). Then, in the second stage, the remaining two actions will be used to select the direction of movement. For the direction of movement of the claw, the first action selected which axis to move along, and the next second action selected to move in the positive or negative direction of that axis. The first actions {REBO, LEBO, TMR}, respectively indicate the selected X, Y, or Z axis. LEb represents the positive direction of the corresponding axis and Bk represents the negative direction. The first action only needs a short duration of about 1 s to ensure that the model can be recognized, but LEb or Bk needs to hold on when the gripper is running. When the action stops, the gripper also stops moving accordingly. For the opening or closing of the gripper, the first facial movement must be TML. When the second action is Bk, it means closing the gripper and the opposite LEb means opening the gripper. When controlling the opening and closing of the gripper, both the first action and the second action only need to last for a short time. After completing the command, the open and closed state of the gripper will be self-locking. In order to prevent the water cup from being erroneously released at high altitude, the gripper opening and closing control is only valid when the gripper is directly above the desktop and the coordinate value of the z-axis is <10 cm. Throughout whole process, the second action {LEb or Bk} is valid only if it is started within 5 s after the first action is successfully recognized or the last non-resting action is itself, otherwise it is invalid and ignored. In Figure 7, when the variable ***State***is 0, it means that it is currently in the first stage; when it is 1, it means that it has entered the second stage from the first stage; and when it is 2, it indicates the state where the second stage continues.

In the third and fourth sessions, the subjects were required to complete drinking task 10 times as in the second part. In these two sessions, the subjects had three attempts to familiarize themselves with the commands of fEMG before starting the task. The difference between the third session and the fourth session is that the third session has a earphone to provide audio feedback to the subjects, while the fourth session has no audio feedback. The audio feedback in the third session mainly announces the intention of the participants after the action recognition. Participants can confirm whether the recognition results are consistent with their own intentions according to the broadcast content in order to adjust the control strategy in time. The content of the broadcast is the upcoming or ongoing movement of the gripper, as shown in Table 2, and he corresponding intent direction is shown in Figure 6A.

**Table 2:** Broadcast contents in audio feedback after the first action or the second action is recognized.

**First movement**	**Broadcast content**	**Second movement**	**Broadcast content**
REBO	Left or right	LEb	Left
		Bk	Right
LEBO	Forward or back	LEb	Back
		Bk	Forward
TMR	Up or down	LEb	Up
		Bk	Down
TML	Gripper	LEb	Close
		Bk	Open

## 4. Results

### 4.1. Offline Performance

**Figure 8:**
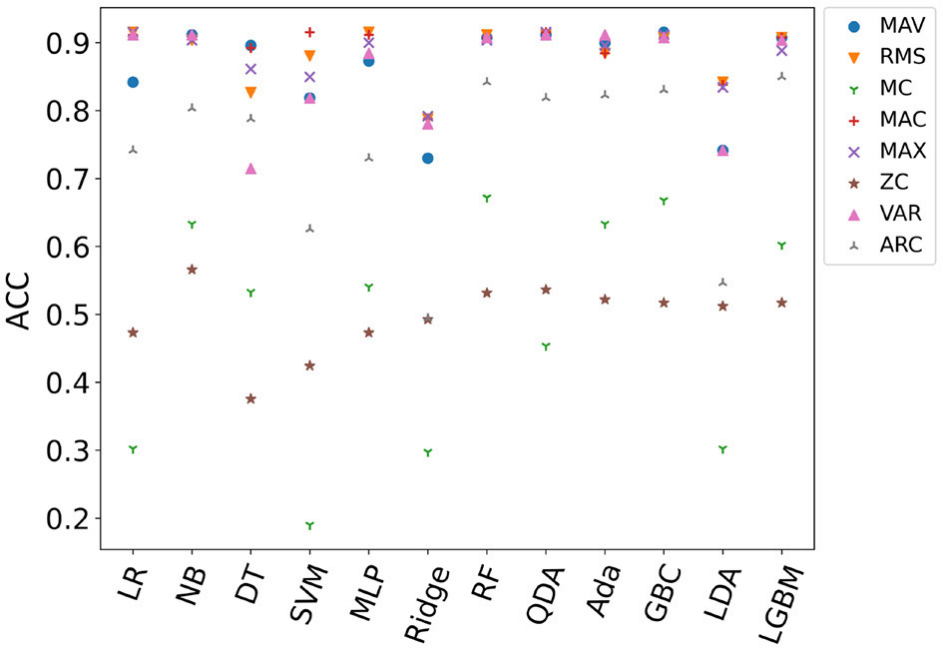
Comparison of the average accuracy of all subjects in different models with single-feature (SF).

**Figure 9:**
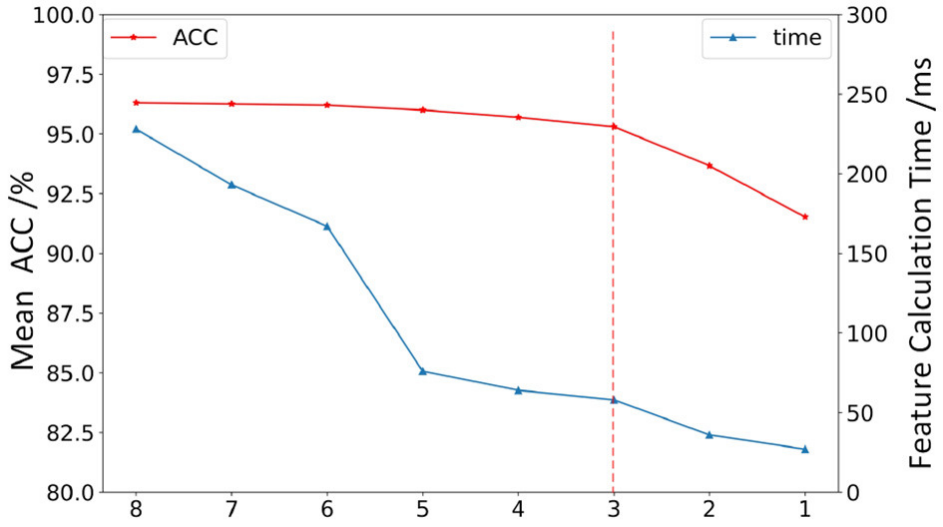
The features calculation time and the average accuracy of the GBC model under feature reduction process. The abscissa represents the performance when the eight features are removed one by one in the order of {MC, ZC, ARC, WAV, MAX, VAR, RMS, MAC}.

Figure 8 shows the average accuracy of all subjects in different models with single-feature (SF). It can be seen from the result that the maximum accuracy of classifiers {LR, NB, SVM, MLP, RF, QDA, Ada, GBC, LGBM} exceeds 90% when using SF as input. Features {MAV, RMS, MAC, MAX, VAR} performed well on multiple classifiers. The features used in the online phase require less time for feature calculation and high recognition accuracy. In order to select the feature set that meets this condition, the average accuracy of all models when a single feature is used as the model input is sorted as {MC, ZC, ARC, WAV, MAX, VAR, RMS, MAC} from low to high. Figure 9 shows the calculation time and the average accuracy of the GBC model under feature reduction process when the eight features are removed one by one in the order of {MC, ZC, ARC, WAV, MAX, VAR, RMS, MAC}. It can be seen from the figure that as the features decrease, the accuracy and calculation time are also decreasing. However, the degree of accuracy decrease in the early stage is small, and the decrease rate increases in the later stage, and its inflection point appears when there are only three features left and the calculation time is already small enough at this time. Therefore, features {VAR, RMS, MAC} are used as EF here.

**Figure 10:**
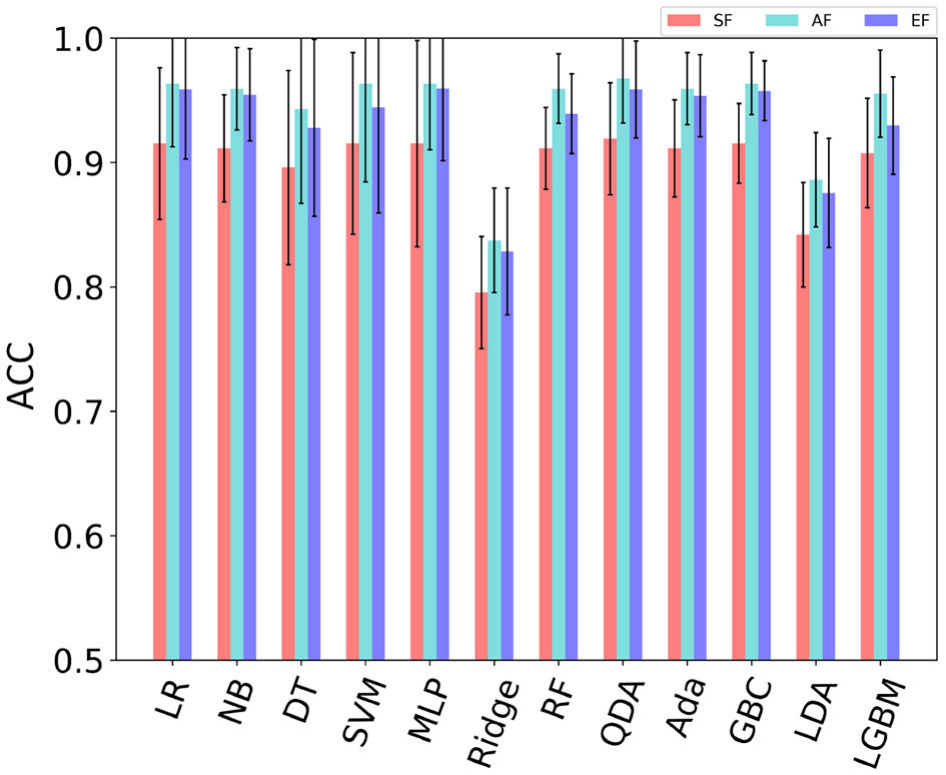
Comparison of the average accuracy of all subjects in different combinations of features. The result is the average of all subjects, and the standard deviation is indicated in the figure. SF: The maximum accuracy rate of each model are selected first across different single-feature (SF) for per single subject, and then the average value of the selected maximum is calculated across all subjects. AF: Performance when using all features. EF: Elected features, here are RMS, MAC, and VAR.

**Figure 11:**
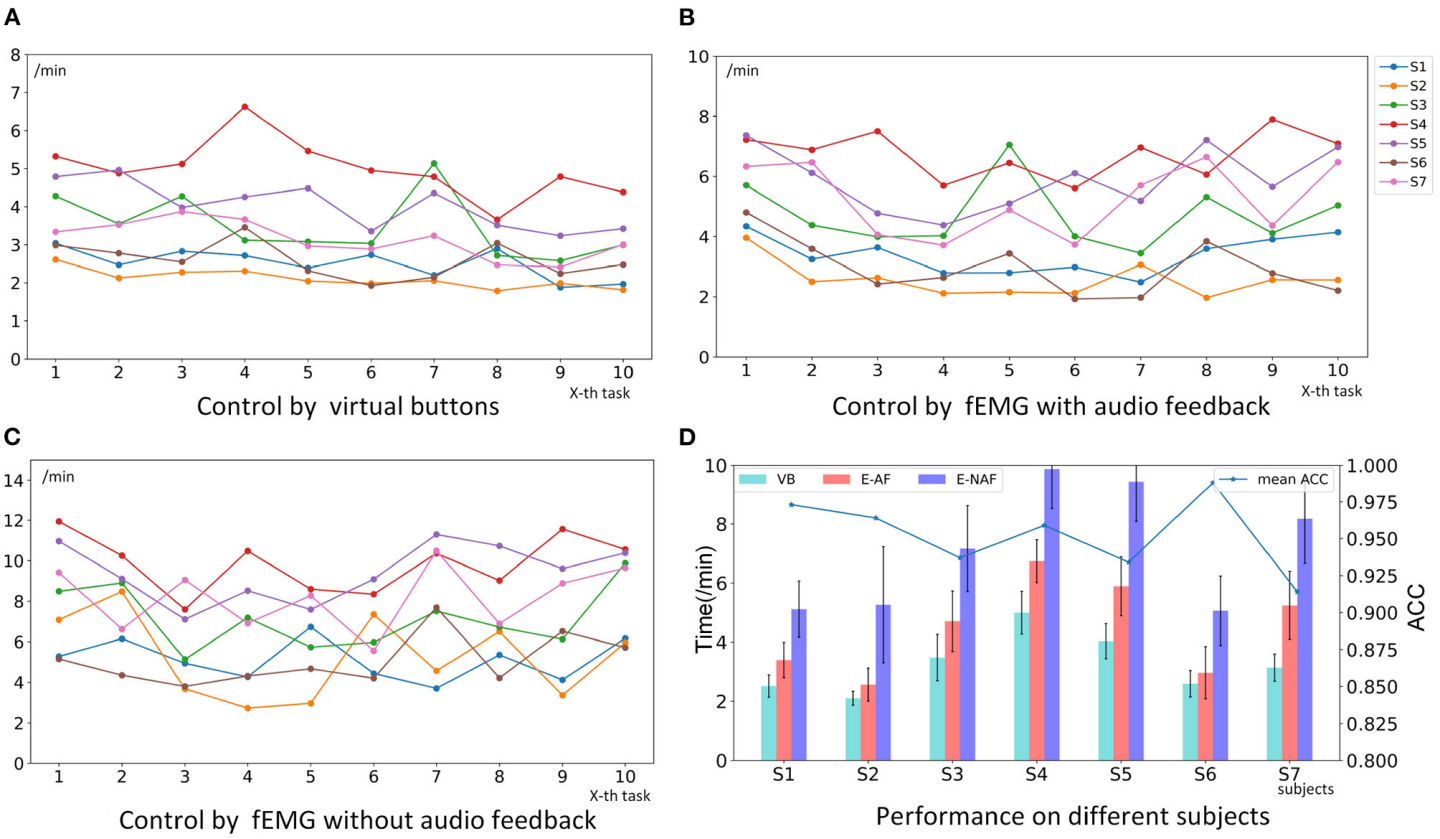
The performance of different subjects using different methods to control the robotic arm to complete the task of drinking water online. **(A)** Control by virtual buttons (VB). **(B)** Control by fEMG with audio feedback (E-AF). **(C)** Control by fEMG without audio feedback (E-NAF). **(D)** Performance on different subjects. The bars represent the average time-consuming of 10 online tasks, and the broken line represents the average recognition accuracy (ACC) of 5 cross-validation using Gradient Boosting Classifier (GBC) with elected features (EF) {RMS, MAC, VAR} as input when offline.

Comparison of the average accuracy of all subjects between SF, AF, and EF is shown in Figure 10. For SF, the maximum accuracy rate of each model are selected first across different single-feature (SF) for per single subject, and then the average value of the selected maximum is calculated across all subjects. The *t*-test was used to analyze the difference between SF and AF, and all *p*-values were <0.01 for all models, which indicates that the performance of SF and AF is significantly different. But there is no statistically significant difference between EF and AF. In other words, the difference between EF and AF is not obvious. When using AF as the model input, there are nine models {LR, NB, SVM, MLP, RF, QDA, Ada, GBC, LGBM } with an average accuracy of more than 95% on all subjects. Among them, the accuracy of 6 models {LR, NB, MLP, QDA, Ada, GBC, LGBM} still exceeds 95% when using EF {RMS, MAC, VAR}. When using AF, the accuracy of QDA is the largest among all models, which is 96.7%. The next one comes from GBC, which is 96.3%. When using EF, the accuracy of MLP is the largest among all models, which is 95.9%. The next one is also from GBC, which is 95.3%. It can be seen that the difference between the best performance when using AF and EF is small, not more than one percentage point. At the same time, the smallest standard deviation of the accuracy comes from GBC in all models. This indicates that GBC has the most stable performance. As a result, GBC with EF input was selected for online testing. When using the EF features{RMS, MAC, VAR} as the input of the GBC model, the average value of the five cross-validation for each participant is shown in Figure 11D. Among them, the accuracy of subject S6 was the highest with 98.8%.

### 4.2. Online Control Robotic Arm

The performance of different subjects using different methods to control the robotic arm to complete the task of drinking water online is shown in Figure 11. The time spent by seven subjects using virtual buttons to control the robotic arm to complete the task ranges from 1.79 to 6.62 min. Where the mean is 3.26 min, and the median is 3.02 min. The ranges of the time of using FCI with audio and without feedback are [1.93, 7.9] and [2.18, 9.55] min, respectively. Where the means are 4.5 and 5.72 min, and the medians are 4.13 and 5.60 min. In terms of mean and variance, the performance using virtual buttons as the control method is better than fEMG control with audio feedback, and the performance without audio feedback is the worst. For these three cases, the pairwise permutation test was used to analyze their statistical significance. Ten thousand permutation tests were carried out for each condition of each subject, and the *p*-value is shown in Table 3. As can be seen from the table, for the control by virtual buttons or by fEMG with audio feedback, the significance of the difference between each subject is quite different. S6 has no statistical difference, S1, S2, and S3 have a certain difference, while the difference of S4, S5, and S7 is very significant. Therefore, statistically speaking, there are some differences between virtual buttons and fEMG with audio feedback, but the overall difference is not very significant. For the control by fEMG with and without audio feedback, the *p*-values of all subjects are <0.001, indicating that the difference between this two cases is very statistically significant. The average time of each participant in the 10 tasks is marked in Figure 11D. It can be seen that there is no order of magnitude difference in the time consumed by virtual buttons and fEMG with audio feedback. The average gap between the two is 1.24 min. The biggest gap comes from the subject S7, which is 2.1 min, and the smallest difference is only 0.37 min which is from S6.

**Table 3:** In the online phase, the *p*-value of the three-way pairwise permutation test statistical analysis.

**Pair**	**S1**	**S2**	**S3**	**S4**	**S5**	**S6**	**S7**
VB & E-AF	0.0015	0.019	0.008	0.0002	0.0004	0.28	<0.0001
VB & E-NAF				All <0.0001
E-AF & E-NAF	0.0003	0.0005	0.0007	<0.0001	<0.0001	0.0003	0.0004

*Among them, VB, control by virtual buttons; E-AF, control by fEMG with audio feedback; E-NAF, control by fEMG without audio feedback*.

## 5. Discussion

This study performed offline analysis and online experiment respectively based on the proposed FCI. In the offline stage, 12 models and eight ways of calculating features were compared in detail. A total of seven participants performed 25 rounds of fEMG signal acquisition for six facial movements, and each generated 20 rounds of valid signals. It can be seen from the offline analysis results that the selected action has good recognizability under the studied model and features. As shown in Figures 10, 11D, when the EF features {RMS, MAC, VAR} were selected as the model input, the maximum recognition accuracy among the seven participants reached 98.8%, and the minimum reached 91.4%. In the feature calculation methods studied, the time domain features were mainly compared without the frequency domain features. Because the main purpose of comparing features is to select some good features for online use, and these features selected usually require fast and stable calculations. When calculating features related to the frequency domain, the speed is usually slower than that in the time domain. Moreover, it can be seen from the offline and online results that the recognition accuracy and efficiency are sufficient based on EF features {RMS, MAC, VAR} or even a single feature only.

In order to verify the effectiveness of the proposed FCI, an experiment to control a robotic arm was designed. During the online control of the robotic arm, four progressive sessions were carefully designed. In the experiment, in order to reduce the burden on the participants and keep FCI close to the daily habits of most people, participants only learned the motion control of the gripper without having to understand the working principle of the robotic arm too much. This way is as natural as people controlling arbitrary objects to move in three-dimensional space in normal life. Subjects first used the virtual buttons on the touch screen to control the gripper to perform the task of drinking water, and then used FCI to control the robotic arm. A total of six facial actions from almost entire face in the online phase were used to map to the eight movements commands of the grippers of end at robotic arm. When the virtual buttons, fEMG with audio feedback and fEMG without audio feedback were used to control the hand grips, the performance on the virtual buttons was the best, which was reasonable and expected. Using the button control method is just a benchmark, or even an upper limit that can be reached at present. This method is only suitable for scenarios where hand functions are available. In the case of lack of limb functions, this method cannot continue to be used. In contrast, the FCI method with relatively poor performance but not very large gap is more suitable for disabled people.

It can be seen from the results of the online stage that the movements recognition accuracy is not the only factor that affects the performance of the experiment. There are many factors that affect the performance of the online control stage. These factors include: (1) the accuracy of intention recognition; (2) whether the feedback information is sufficient, such as whether there is voice feedback; (3) whether the actions of the participants are consistent with their intentions; (4) the participant's reaction speed, such as the time it takes to correct the movement or recognition error; (5) the participant's path planning and control strategy for the movement of the gripper (Nam et al., 2014; Zhang et al., 2020). As shown in Figure 11, the accuracy rate from subjects S3, S5, or s2 was lower than that from S4, but in the online process, the former performed better than the latter. S6 had the highest accuracy rate reached 98.8%, but his (/her) time-consuming performance was not the best, slightly inferior to S2. Similarly, the accuracy of S7 was the lowest, but the performance of online control was not the worst. There is a trend that the more time the subjects spend based on virtual buttons, the more time they spend based on FCI. The performance of subjects S1, S2, and S6 under using FCI was even higher than that of subjects S3, S4, and S5 from using virtual buttons. Therefore, if the time subjects take to use the buttons to control the robotic arm to complete the task of drinking water is acceptable, then the time spent using FCI should also be within an acceptable range.

For fEMG-based FCI, the method with audio feedback performed better than that without audio feedback. When there is audio feedback, not only did the participants spend less time to complete the task, but their performance was also more stable. It can be seen from Figures 11B,C that, when there is no audio feedback, the time spent on 10 tasks fluctuates more severely. Figure 11D also shows the standard deviation of the time it takes on different task trials. It can also be seen that the standard deviation when there is no voice feedback is larger than when there is voice feedback.

FCI based on fEMG has some inherent disadvantages, such as the need to attached electrodes on the subject's face, this may not be accepted by some people. However, this requirement is more suitable than EEG-based HCI that requires EEG caps and conductive paste. In addition, like other HCI based on physiological signals, FCI based on fEMG also requires additional signal acquisition for model training. And fatigue caused by long-term continuous use will reduce performance without a lot of corresponding exercise. Compared with a button-based HCI, FCI based on fEMG also has some advantages. One of the advantages is that the user can keep his eyes on the movement of the object to be controlled without leaving it when using FCI. However, when using HCI that is operated by hand based on buttons or joysticks, the subjects often need to shift their attention to their hands first when switching commands, and the sight of the subjects even jumped back and forth between the hand and the object to be controlled sometimes.

## 6. Conclusion

A complete FCI framework based on fEMG including offline analysis and online control of mechanical equipments was proposed. In the offline stage, 12 models, eight ways of calculating features, and three ways of feature input were studied and compared in detail. The three EF features {RMS, MAC, VAR} and the GBC model with an average offline recognition rate of 95.3%, a maximum of 98.8%, and a minimum of 91.4% were selected for use in online scenarios. Four well-designed sessions were designed for online verification and performance comparison of FCI. In the online phase, seven subjects were required to use virtual buttons, fEMG with and without audio feedback to control the gripper at the end of the robotic arm to complete the drinking experiment. In contrast, the way with audio feedback performed better than the way without audio feedback. There is no order of magnitude difference in the time consumed by virtual buttons and fEMG with audio feedback. The average gap between the two is only 1.24 min, and the smallest difference is only 0.37 min. The effectiveness and applicability of the proposed FCI framework has been proven.

## Data Availability Statement

The original contributions presented in the study are included in the article/supplementary material, further inquiries can be directed to the corresponding author/s.

## Ethics Statement

The studies involving human participants were reviewed and approved by the Ethical Committee of the Shenyang Institute of Automation. The patients/participants provided their written informed consent to participate in this study. Written informed consent was obtained from the individual(s) for the publication of any potentially identifiable images or data included in this article.

## Author Contributions

BZ and YC carried out experimental design, data collection, analysis, and wrote the first draft of this article. LZhan and LZhao provided anatomical analysis support, corrected the electrode placement position, and designed the main types of movements used. DZ provided technical guidance in online experiments. XZ provided the experimental platform and its management and completed the final proofread. All authors contributed to the article and approved the submitted version.

## Conflict of Interest

The authors declare that the research was conducted in the absence of any commercial or financial relationships that could be construed as a potential conflict of interest.

## Abbreviations

Base: FCI: face-computer interface
fEMG: facial electromyography
HCI, human-computer interface. Facial movements: LEb: lift eyebrows
LEBO: left eye blink once
REBO: right eye blink once
Bk: bick
TML: tilt mouth to left
TMR, tilt mouth to right. Models: LR: logistic regression
NB: Naive Bayes
DT: decision tree
SVM: support vector machines with Linear Kernel
MLP: multilayer perceptron
Ridge: ridge classifier
RF: random forest
QDA: quadratic discriminant analysis
Ada: Ada boost
GBC: gradient boosting classifier
LDA: linear discriminant analysis
LGBM, light gradient boosting machine Features: MAV: mean absolute value
RMS: root mean square
MC: mean changes
MAC: mean absolute changes
MAX: maximum value
ZC: zero crossings
VAR: variance
ARC, auto regression coefficient. Feature combinations: SF: single-feature
AF, all-features. Online control: VB: control by virtual buttons
E-AF: control by fEMG with audio feedback
E-NAF: Control by fEMG without audio feedback.
